# Effective Dose of *Rhizoma Coptidis* Extract Granules for Type 2 Diabetes Treatment: A Hospital-Based Retrospective Cohort Study

**DOI:** 10.3389/fphar.2020.597703

**Published:** 2021-01-25

**Authors:** Yueh-Hsiang Huang, Geng-Hao Liu, Tzu-Yang Hsu, Lan-Yan Yang, Ming-Chung Lee, Chun-Teng Huang, Yi-Hong Wu

**Affiliations:** ^1^ Division of Chinese Internal Medicine, Center for Traditional Chinese Medicine, Chang Gung Memorial Hospital, Taipei, Taiwan; ^2^ Graduate Institute of Clinical Medical Sciences, College of Medicine, Chang Gung University, Taoyuan, Taiwan; ^3^ School of Traditional Chinese Medicine, College of Medicine, Chang Gung University, Taoyuan, Taiwan; ^4^ Division of Acupuncture and Moxibustion, Department of Traditional Chinese Medicine, Chang Gung Memorial Hospital, Linkou, Taiwan; ^5^ Biostatistics and Informatics Unit, Clinical Trial Center, Chang Gung Memorial Hospital and Chang Gung University, Taoyuan, Taiwan; ^6^ Brion Research Institute of Taiwan, New Taipei, Taiwan; ^7^ Sanford Burnham Prebys Medical Discovery Institute, La Jolla, CA, United States

**Keywords:** effective dose, Chinese herbal medicine, type 2 diabetes, extract granules, *Rhizoma coptidis*

## Abstract

*Rhizoma Coptidis* is a popular phytomedicine for the treatment of type 2 diabetes in Asia, but its effective dose for diabetes treatment remains confused because of diverse origins. This study aimed to investigate the dose-response effects of *Rhizoma Coptidis* extract granules (RCEG), produced with standardized quality control, on hypoglycemic effects in patients with type 2 diabetes. We conducted a retrospective analysis of Chang Gung Research Database from January 01, 2008 to November 30, 2017. Outpatients visiting traditional Chinese medicine clinics and receiving RCEG for type 2 diabetes treatment were included. Plasma glucose, lipid, and other parameters were analyzed from 93 patients with a total of 737 visits within 60 weeks. Scatter plots with the LOESS analysis were used to explore the association between RCEG dose and hypoglycemic effect. The minimal effective dose was chosen to divide the study population into the high-dose and low-dose RCEG groups. Non-parametric tests were used for between-group and within-group comparisons. The multivariate nonlinear mixed-effects model was applied to access the effect of treatment length and groups simultaneously on the change of HbA1c and fasting plasma glucose. The “arule” package in R was used to present the network diagram of RCEG and other co-prescriptions. We first discovered a significant relationship between RCEG dose and HbA1c reduction when the dose reached 0.08 g/kg/day or higher. We thus defined 0.08 g/kg/day of RCEG as the minimum effective dose, and a threshold to separate patients into the high-dose (≥0.08 g/kg/d) and low-dose (<0.08 g/kg/d) RCEG groups. In the high-dose RCEG group, a significant decrease in total cholesterol and a trend toward triglyceride reduction were also noted. Patients more effectively responded to RCEG treatment if they had a higher initial HbA1c level, higher heart rates, better liver function tests, and better tolerance to the higher dose and treatment duration of RCEG. In addition, digestive/tonic/dampness draining formulas and blood regulation recipes were two of the most frequent co-prescriptions with RCEG. This study concluded that RCEG at a dose exceeding 0.08 g/kg/d had beneficial effects on glycemic and lipid control, without showing nephro- or hepatotoxicity, in patients with type 2 diabetes.

## Introduction

*Rhizoma Coptidis*, commonly known as Huang-lian, was first recorded in *Shennong’s Materia Medica* in the eastern Han dynasty (25–220 AD), and it has been prescribed to treat diabetes-related symptoms “Xiaoke (消渴)” for the past 2000 years. According to traditional Chinese medical theory, *Rhizoma Coptidis* is cold in nature with bitter in taste, and has the function of purging fire toxins, clearing heat, and drying dampness. The phytochemical and therapeutic activities of *Rhizoma Coptidis* have been extensively reviewed in the literature (Wang et al., 2014; Pang et al., 2015a; Ma et al., 2019; Wang et al., 2019), and *Rhizoma Coptidis* is an excellent source of phytomedicine for treating type 2 diabetes. In Taiwan, most traditional Chinese medicine (TCM) doctors prescribe *Rhizoma Coptidis* in the form of extract granules instead of herbal decoctions, because the former is covered by national health insurance. In addition, for Chinese herbal medicines (CHM), granule is the most prominent delivery format in the West. *Rhizoma Coptidis* extract granules (RCEG) produced by good manufacturing practice (GMP) manufacturers are available in many countries, including the United States, Canada, Switzerland, Malaysia, Singapore, and some European countries. Although *Rhizoma Coptidis* is widely prescribed for many diseases, including type 2 diabetes (Meng et al., 2018), the effective dose and dose-response relationship of RCEG for diabetes treatment remain poorly understood (Wang et al., 2014).

The global prevalence of diabetes mellitus is rising rapidly, and it has become a major public health issue worldwide (Ogurtsova et al., 2017). In Taiwan, the increasing prevalence of diabetes is in line with the global trend (Sheen et al., 2019). Only 32.4% of patients with diabetes achieve the American Diabetes Association (ADA) goal to control their hemoglobin A1c (HbA1c) level below 7% (Yu et al., 2009). According to a previous national population-based study in Taiwan, single herbal medicines commonly prescribed for diabetes treatment were not reported (Huang et al., 2013). Instead, a formula called Liu-Wei-Di-Huang-Wan or its derivatives were the most common prescriptions to treat patients with diabetes (Huang et al., 2013), even though the hypoglycemic effect of Liu-Wei-Di-Huang-Wan treatment alone for type 2 diabetes has not been tested in rigorous high-quality trials (Lin et al., 2016; Zheng et al., 2020). In fact, there is no information about CHM and its anti-diabetes-related biochemical parameters in the national health insurance research database (NHIRD) of Taiwan, and the accuracy of the diagnostic coding process is often criticized. Therefore, it is challenging to investigate the effect of CHM on glycemic control in diabetes based on the NHIRD.

The Chang Gung Research Database (CGRD) is the largest multi-institutional, electronic medical record database in Taiwan (Tsai et al., 2017; Shao et al., 2019). Diagnosis reports of patients with type 2 diabetes are confirmed by laboratory exams and archived in the CGRD. In this retrospective study, medical records of patients with type 2 diabetes prescribed with RCEG from TCM clinics in the Taipei, Linkou, and Taoyuan branches of Chang Gung Memorial Hospital (CGMH) were retrieved from the CGRD. The goal of this study is to investigate the dose-response effect of RCEG on glycemic control, and determine the minimum effective dose as a threshold to distinguish the high-dose from the low-dose RCEG group. In addition to the anti-diabetic effects, other therapeutic effects, safety parameters, and factors affecting the drug response were examined. We also explored other CHM extract granules commonly co-prescribed with RCEG to treat type 2 diabetes. This study provides a rationale for TCM doctors to prescribe RCEG for patients with type 2 diabetes, and serves as a stepping stone for future clinical trials.

## Methods

### Study Population

**Figure 1:**
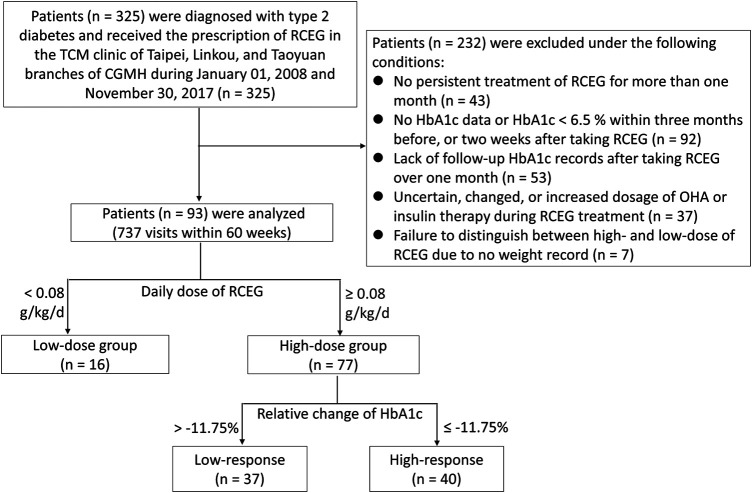
Flowchart of recruitment from the Chang Gung Research Database. CGMH, Chang Gung Memorial Hospital; HbA1c, glycated hemoglobin; OHA, oral hypoglycemia agents; RCEG, *Rhizoma Coptidis* extract granules; TCM, traditional Chinese medicine.

The Ethics Review Board of Chang Gung Medical Foundation in Taiwan approved this research (IRB No. 201701817B0). All data were collected from the CGRD and encrypted to protect the privacy of the patients. Medical records of outpatients who were diagnosed with type 2 diabetes and received the prescription of RCEG in the TCM clinics of Taipei, Linkou, and Taoyuan branches of Chang Gung Memorial Hospital from January 01, 2008 to November 30, 2017 were included. Diagnosis codes for type 2 diabetes include 250.00, 250.02, 250.40, 250.42, 250.50, 250.52, 250.60, 250.62, 250.80, 250.82, 250.90, and 250.92 in the International Classification of Disease, 9th Revision, Clinical Modification (ICD-9-CM) format, or E11.8, E11.9, E11.65, E11.69, E11.29, and E11.21 in the ICD-10-CM format. Patients were excluded from this study based on the following criteria: 1) no persistent treatment with RCEG for more than one month; 2) no HbA1c data or HbA1c <6.5% within three months before or two weeks after taking RCEG; 3) lack of HbA1c follow-up after taking RCEG for over one month; 4) uncertain, changed, or increased dosage of oral hypoglycemic agents (OHA) or insulin therapy during RCEG treatment; 5) failure to distinguish between high- and low-dose of RCEG due to no weight being recorded. The flowchart of the recruitment process is presented in Figure 1.

### *Rhizoma Coptidis* Extract Granules (RCEG)

The RCEG prescribed in this study were manufactured by Sun Ten Pharmaceutical Co., Ltd., with standardized quality control. The botanical origin of RCEG was the dried rhizome of *Coptis chinensis Franch*. The chemical composition of the RCEG was analyzed and profiled by using high performance liquid chromatography (HPLC) with photodiode array (PDA) detection (Supplementary Material S1). The batch numbers of RCEG during the study period were also recorded (Supplementary Material S1). In terms of quantitative detection, berberine is the indicative compound of *Rhizoma Coptidis*. Each Gram of RCEG contained no less than 42 mg of berberine, according to the standard requirement of the Taiwan Herbal Pharmacopeia (Ministry of Health and Welfare, 2016).

### Data Collection and Outcome Measurement

Data collection from the CGRD included the following information: 1) diagnosis codes; 2) date of visit; 3) name of CHM, dosage, frequency of administration, and treatment duration; 4) name and dose of OHA and insulin; 5) date and data of laboratory tests; 6) general information, including age, gender, and duration of diabetes; 7) body weight and height; 8) blood pressure and heart rate. There were very few patients receiving RCEG treatment for more than 60 weeks, so this study gated the maximum follow-up period to 60 weeks. Patients may have had different follow-up times because of the differing severity of their symptoms, drop outs, discontinuous RCEG treatment, or lack of laboratory data. The hypoglycemic response to RCEG treatment referred to changes of HbA1c level from pre-treatment to each patient’s last treatment. Scatter plots and the LOESS method were used to explore the association between RCEG dose and hypoglycemic effect in order to determine an optimal cutoff value for the dosage, thus allowing the study population to be divided into high-dose and low-dose RCEG groups for further analysis. The baseline characteristics of these two groups were analyzed. During RCEG treatment, the changes in HbA1c and fasting blood glucose at different time points were analyzed in both groups. The relative change was calculated by dividing the net change by the baseline value. Particularly in the high-dose group, the effect of treatment was measured based on the value changes from pre-treatment to the last available post-treatment due to limited data in some parameters. Co-prescription patterns and the daily dose of other CHM extract granules commonly combined with high-dose RCEG were also accessed. For each outpatient visit, the details of prescriptions, including extract granules of herbal formulas and single herbs, were recorded. In this study, a total of 636 prescriptions with high-dose RCEG were assessed. Frequent itemsets mining and rules of association were applied to evaluate the co-prescription patterns of CHM extract granules combined with RCEG. In the high-dose group, the relative change between the lowest HbA1c value and the first measured HbA1c value for each patient was calculated. The median relative change of HbA1c in the 12th week was –11.75%, and this value was set as a cutoff threshold to distinguish the high-response and low-response population within the high-dose RCEG group. The baseline characteristics, average daily dose and treatment duration of RCEG, and co-prescription patterns between the high- and low-response groups were analyzed.

### Statistical Analysis

Descriptive statistics were used to summarize the data. The relationship between RCEG dose and relative change of HbA1c was presented in the form of scatter plots with the LOESS analysis. Comparisons between groups were performed with the Mann-Whitney U-test for continuous parameters and Fisher's exact test for categorical parameters. The Wilcoxon signed rank test was used for pre- and post-comparison within the groups. The multivariate nonlinear mixed-effects model was applied to access the effect of treatment length and groups simultaneously on the change of HbA1c and fasting plasma glucose (FPG). The “arule” package in R was used to explore the common CHM combinations and present the CHM network in this study. A value of *p* < 0.05 was considered as the significance threshold for all two-tailed tests.

## Results

### Baseline Characteristics of the High- and Low-Dose RCEG Groups

There were 325 TCM outpatients diagnosed with type 2 diabetes according to the HbA1c ≥ 6.5% criteria and prescribed with RCEG from January 01, 2008 to November 30, 2017. Only 93 patients, who engaged in 737 visits within 60 weeks, were eligible and analyzed in this study (Figure 1). The analysis via the LOESS regression method (Supplementary Material S2) indicated a significant relationship between RCEG dose and HbA1c reduction when the dose was above 0.08 g/kg/day. We therefore suggested that 0.08 g/kg/day is the minimum effective dose of RCEG to treat type 2 diabetes, and it was used as the local optimal cutoff to group patients into a high-dose (≥0.08 g/kg/d) RCEG population (*n* = 77) and low-dose (<0.08 g/kg/d) RCEG population (*n* = 16). Baseline characteristics, including demographic records, anthropometric characteristics, vital signs, and laboratory data of the high-dose and low-dose RCEG groups are shown in Table 1. There were no significant differences between the two groups with respect to age, gender, use of hypoglycemic drugs or insulin, anthropometric characteristics, hepatic and renal function, and lipid profiles. In the high-dose group, the median duration of diabetes was 8 years and the median HbA1c level before RCEG treatment was 8.4%. Both median values were significantly higher than 3 years of diabetes duration and 7.2% of HbA1c in the low-dose group.

**Table 1:** Baseline characteristics of the high-dose and low-dose RCEG groups.

	High-dose group	(*n* = 77)	Low-dose group	(*n* = 16)	*p*-value
	Median (min, max)		Median (min, max)		
Demographic characteristics					
Age (years)	60 (41, 81)	(*n* = 77)	62 (45, 78)	(*n* = 16)	0.33
Gender, male/Female	41/36	(*n* = 77)	10/6	(*n* = 16)	0.59^a^
Duration of DM (years)	8 (1, 28)	(*n* = 77)	3 (1, 11)	(*n* = 16)	0.0095**
No. Of OHA (0/1/2/3/4)	35/11/11/15/5	(*n* = 77)	13/2/1/0/0	(*n* = 16)	0.10^a^
Insulin used (Y/N)	6/71	(*n* = 77)	1/15	(*n* = 16)	1.00^a^
Anthropometric characteristics and vital signs					
Weight (kg)	66.8 (40.0, 121.6)	(*n* = 77)	67.0 (47.0, 88.9)	(*n* = 16)	0.99
BMI (kg/m2)	25.6 (16.2, 41.6)	(*n* = 76)	26.1 (20.7, 35.4)	(*n* = 15)	0.63
Height (cm)	163.5 (140.0, 178.0)	(*n* = 77)	164.4 (146.5, 175.1)	(*n* = 16)	0.75
SBP (mmHg)	136.0 (105.0, 187.0)	(*n* = 68)	129.5 (106.0, 158.0)	(*n* = 14)	0.14
DBP (mmHg)	79.5 (40.0, 112.0)	(*n* = 68)	76.5 (59.0, 106.0)	(*n* = 14)	0.61
HR (beat/min)	81.0 (60.0, 111.0)	(*n* = 59)	81.0 (65.0, 104.0)	(*n* = 14)	0.58
Laboratory data					
HbA1c (%)	8.4 (6.5, 13.9)	(*n* = 77)	7.2 (6.5, 10.9)	(*n* = 16)	0.001**
FPG (mg/dl)	166.0 (83.0, 353.0)	(*n* = 39)	132.5 (103.0, 239.0)	(*n* = 14)	0.08
2 hPG (mg/dl)	247.0 (96.0, 436.0)	(*n* = 22)	121.5 (111.0, 132.0)	(*n* = 2)	0.18
ALT (U/L)	27.0 (9.0, 106.0)	(*n* = 45)	35.0 (13.0, 138.0)	(*n* = 10)	0.50
Cr (mg/dl)	0.7 (0.3, 8.0)	(*n* = 46)	0.8 (0.6, 4.3)	(*n* = 11)	0.87
Total cholesterol (mg/dl)	180.0 (86.0, 384.0)	(*n* = 39)	176.6 (98.0, 210.0)	(*n* = 14)	0.88
HDL-C (mg/dl)	42.0 (26.0, 106.0)	(*n* = 36)	46.0 (28.0, 57.0)	(*n* = 13)	0.98
LDL-C (mg/dl)	102.0 (20.0, 202.0)	(*n* = 37)	108.0 (20.0, 137.0)	(*n* = 12)	0.72
Triglycerides (mg/dl)	130.0 (33.0, 2,589.0)	(*n* = 39)	124.5 (62.0, 237.0)	(*n* = 14)	0.58

^a^ Fisher’s exact test was used. Statistical significance (**p < 0.01) was shown. ALT, alanine aminotransferase; BMI, body mass index; Cr, creatinine; DBP, diastolic blood pressure; DM, diabetes mellitus; FPG, fasting plasma glucose; HbA1c, glycated hemoglobin; HDL-C, high-density lipoprotein cholesterol; HR, heart rate; LDL-C, low-density lipoprotein cholesterol; OHA, oral hypoglycemia agents; 2 hPG, 2-hour postprandial glucose; SBP, systolic blood pressure.

### Anti-Diabetes and Anti-hypercholesterolemia Effects in the High-Dose RCEG Group

**Figure 2:**
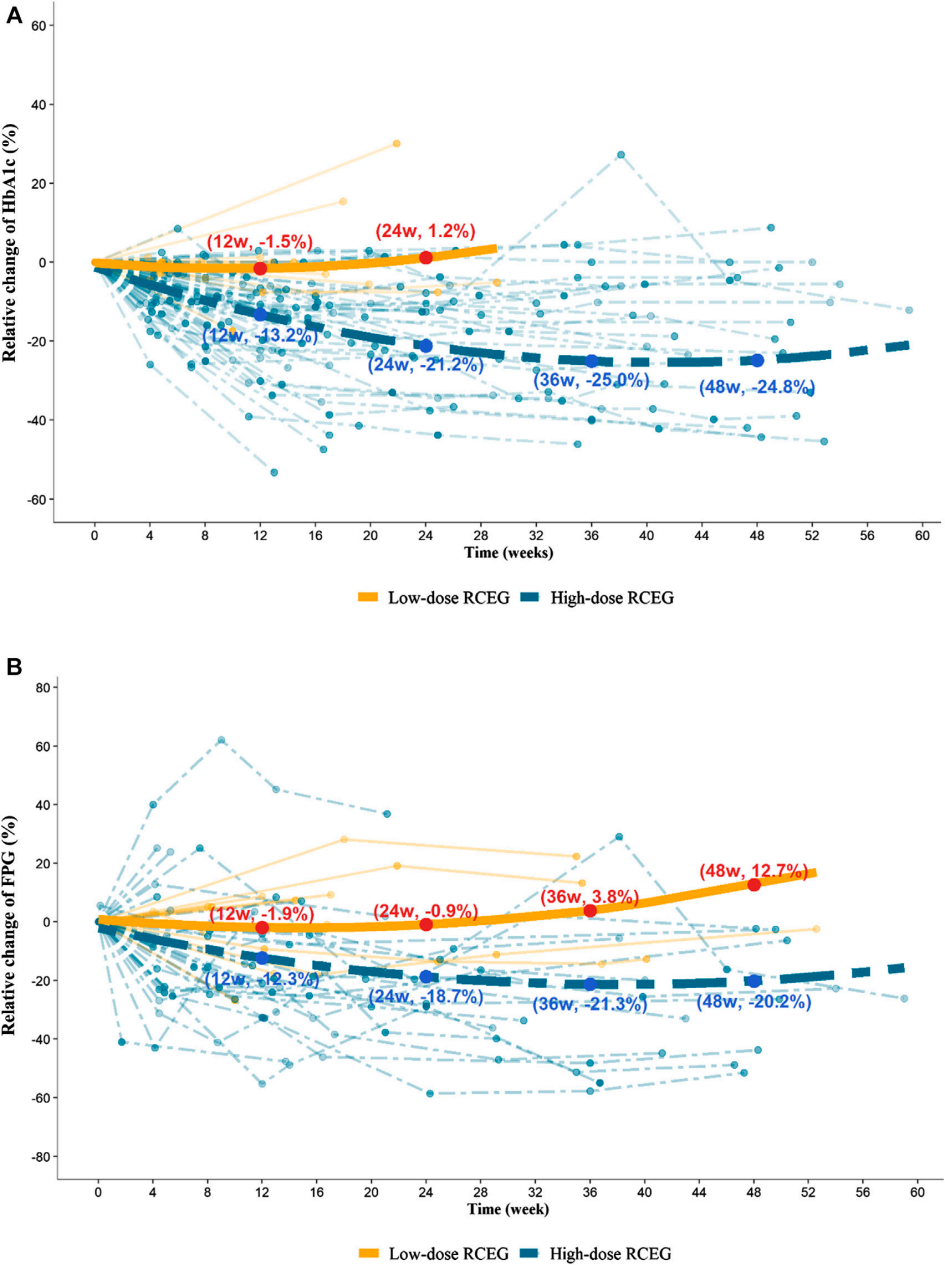
Trajectory plots for the percentage of relative change in HbA1c **(A)** and FPG **(B)** in the high-dose and low-dose RCEG group. Data points presented in aquamarine belong to the high-dose group, and those in orange refer to the low-dose group. Each dashed and solid thin line represents the time-course of HbA1c and FPG changes for a patient in the high-dose and low-dose RCEG group, respectively. A multivariate nonlinear mixed-effects model was applied to acquire the dashed and solid thick lines, exhibiting the fitted mean profiles of patients treated with high-dose and low-dose RCEG, respectively. **(A)** The mean percentage change in HbA1c from baseline at 12, 24, 36, and 48 weeks in the high- and low-dose groups. **(B)** Relative changes in mean FPG at the same time points in both groups. FPG, fasting plasma glucose; HbA1c, glycated hemoglobin; RCEG, *Rhizoma Coptidis* extract granules.

The relative changes in HbA1c and FPG within the 60-weeks treatment period showed significant reductions in the high-dose RCEG group, but not in the low-dose group (Figure 2). In the high-dose group, the mean percentage decrease in HbA1c from baseline was 13.2%, 21.2%, and 25.0% in 12, 24, and 36 weeks, respectively. The mean relative change in FPG from baseline also showed a significant reduction, by 12.3%, 18.7%, and 21.3% at the same time points. The reduction slope of both HbA1c and FPG was steeper in the first 12 weeks of treatment, and then gradually flattened from 12 to 48 weeks in the high-dose group. In contrast, no benefits in glycemic control were found in the low-dose RCEG group at all time points. The comparison of the median values before and after high-does RCEG treatment for each therapeutic parameter is listed in Table 2. High-dose RCEG significantly decreased the median values of HbA1c and FPG level from 8.4 to 7% and 166 to 126 mg/dl, respectively (both *p* < 0.001). There was a decrease in 2hPG from 247 to 162 mg/dl, which was not statistically significant, probably due to the limited sample size. Regarding the lipid profiles, a significant decline in total cholesterol from 180 to 154 mg/dl (*p* = 0.04) and a trend of reduction in triglycerides from 130 to 84 mg/dl (*p* = 0.07) were found after the high-dose RCEG treatment. However, there was no significant change in either high- or low-density lipoprotein cholesterol, possibly because of the sample size. In terms of safety parameters, including blood pressure, hepatic function, and renal function, high-dose RCEG exhibited a slightly beneficial effect on blood pressure control (*p* = 0.02) without nephro- or hepatotoxicity.

**Table 2:** Parameters before and after treatment in the high-dose RCEG group.

	Pre-treatment		Post-treatment^a^		*p*-value	
	Median (min, max)		Median (min, max)					
SBP (mmHg)	136.0 (105.0, 187.0)	(*n* = 68)	131.5 (101.0, 167.0)	(*n* = 56)	0.02*	(*n* = 50)
DBP (mmHg)	79.5 (40.0, 112.0)	(*n* = 68)	76.0 (53.0, 95.0)	(*n* = 56)	0.0008***	(*n* = 50)
HR (beat/min)	81.0 (60.0, 111.0)	(*n* = 59)	84.0 (60.0, 105.0)	(*n* = 54)	0.05	(*n* = 45)
HbA1c (%)	8.4 (6.5, 13.9)	(*n* = 77)	7.0 (5.7, 12.4)	(*n* = 77)	<0.0001***	(*n* = 77)
FPG (mg/dl)	166.0 (83.0, 353.0)	(*n* = 39)	126.0 (69.0, 270.0)	(*n* = 45)	0.0002***	(*n* = 34)
2 hPG (mg/dl)	247.0 (96.0, 436.0)	(*n* = 22)	162.0 (77.0, 347.0)	(*n* = 9)	1.00	(*n* = 8)
ALT (U/L)	27.0 (9.0, 106.0)	(*n* = 45)	24.0 (5.0, 88.0)	(*n* = 40)	0.01*	(*n* = 34)
Cr (mg/dl)	0.7 (0.3, 8.0)	(*n* = 46)	0.7 (0.4, 1.6)	(*n* = 42)	0.02*	(*n* = 36)
Total cholesterol (mg/dl)	180.0 (86.0, 384.0)	(*n* = 39)	154.0 (95.0, 319.0)	(*n* = 25)	0.04*	(*n* = 16)
HDL-C (mg/dl)	42.0 (26.0, 106.0)	(*n* = 36)	38.0 (28.0, 61.0)	(*n* = 23)	0.96	(*n* = 13)
LDL-C (mg/dl)	102.0 (20.0, 202.0)	(*n* = 37)	97.0 (55.0, 133.0)	(*n* = 26)	0.26	(*n* = 16)
Triglycerides (mg/dl)	130.0 (33.0, 2,589.0)	(*n* = 39)	84.0 (41.0, 875.0)	(*n* = 25)	0.07	(*n* = 16)

^a^ Post-treatment value was defined as the latest value recorded within the treatment period. Statistical significance (*p < 0.05; ***p < 0.001) was obtained by the Wilcoxon signed-rank test. ALT, alanine aminotransferase; Cr, creatinine; DBP, diastolic blood pressure; FPG, fasting plasma glucose; HDL-C, high-density lipoprotein cholesterol; HR, heart rate; LDL-C, low-density lipoprotein cholesterol; 2 hPG, 2-hour postprandial glucose; SBP, systolic blood pressure.

### Co-Prescription Patterns and Response Evaluation in the High-Dose RCEG Group

**Figure 3:**
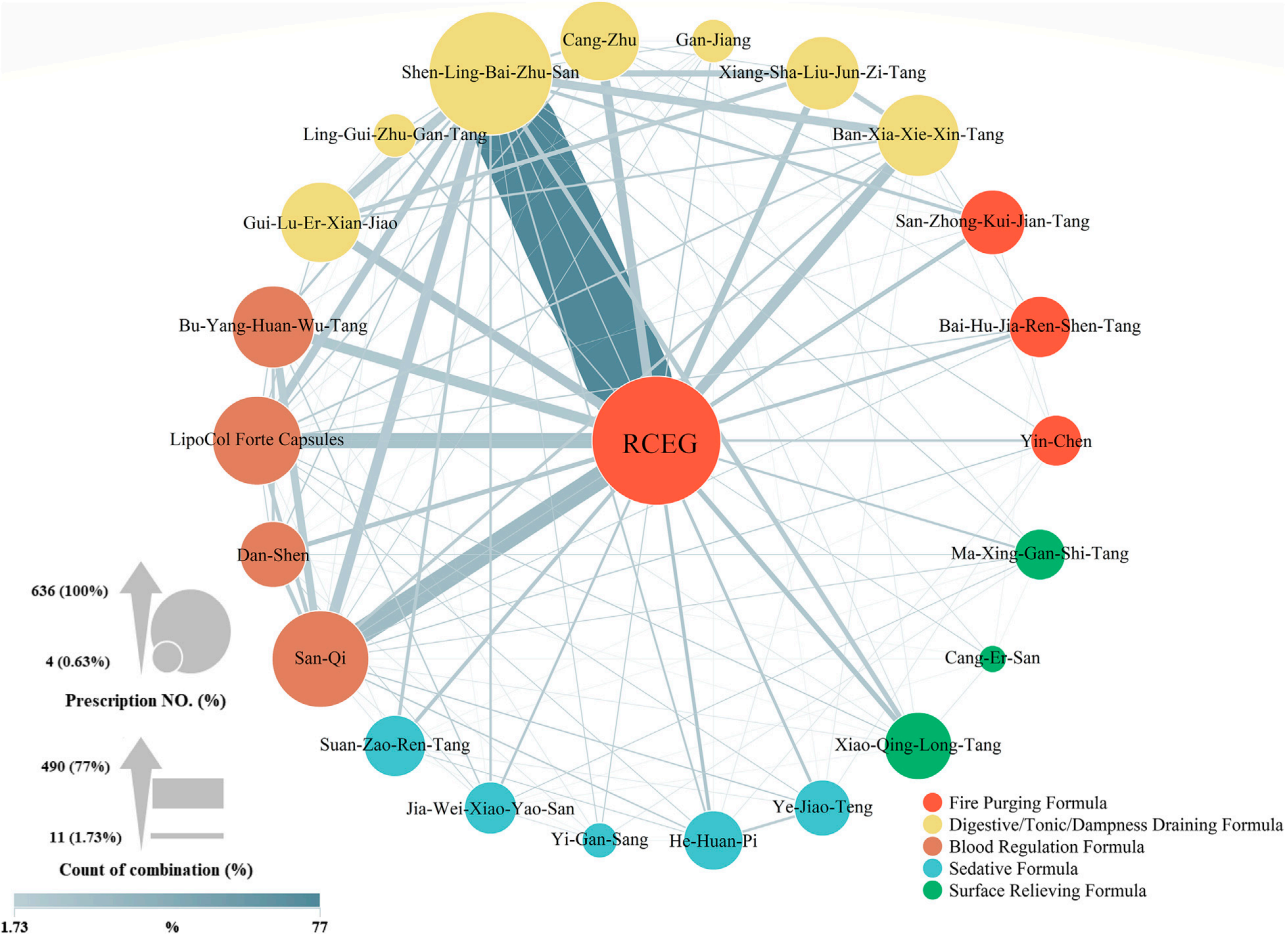
Co-prescription network in the high-dose RCEG group. RCEG, namely Huang-Lian, was plotted in the center of the CHM network as the core prescription for type 2 diabetes in this diagram. The size of a circle is proportional to the prescription frequency of each CHM. The thickness of a connection line between two CHM corresponds to the prevalence of that combination. RCEG, *Rhizoma Coptidis* extract granules; CHM, Chinese herbal medicines.

It is possible that other CHMs, including single herbs, formulas, or a combination of both co-prescribed with RCEG, could influence glycemic control. Therefore, the daily dose of CHM and co-prescription pattern in the high-dose RCEG group were evaluated. The effects and indications of each co-prescribed CHM are summarized in (Supplementary Material S3) (Hempen,and Fischer, 2009; Ministry of Health and Welfare, 2016). Unlike high-dose RCEG, the median daily dose of each CHM was within the general recommended dose. In Figure 3, a network diagram illustrates the co-prescription frequency of CHM with RCEG in the high-dose RCEG group. A larger circle indicates a higher prevalence of a single herb or formula prescribed. A thicker connection line suggests a higher frequency of two CHMs combined. Together with RCEG, Shen-Ling-Bai-Zhu-San (SLBZS) was the most prescribed herbal formula (77.0% of all prescriptions), followed by Ban-Xia-Xie-Xin-Tang (9.8%), Bu-Yang-Huan-Wu-Tang (9.8%), and Gui-Lu-Er-Xian-Jiao (9.0%). In addition, San-Qi (20.4% of all prescriptions), LipoCol Forte Capsules (13.7%), and Cang-Zhu (8.7%) were the top three herbs prescribed with RCEG. The frequently co-prescribed CHMs can be clustered into five distinct categories: fire purging, sedative, surface relieving formulas, digestive/tonic/dampness draining, and blood regulation. The latter two, shown as yellow and brown clusters, were the major combinations in this co-prescription network. Notably, SLBZS and San-Qi were the top co-medications prescribed with RCEG. We further divided the high-dose group into the high-response and low-response populations to identify parameters affecting the glycemic control (Table 3). There was no significant difference in common co-prescription patterns of CHMs, as well as combinations of OHA/insulin therapy, between the high-response and low-response groups. In addition, patients with a better response to the high-dose RCEG had higher initial HbA1c levels, higher heart rates, better liver function tests, and better tolerability to the dosage and duration of RCEG treatment. Surprisingly, Liu-Wei-Di-Huang-Wan and Ba-Wei-Di-Huang-Wan, the two most commonly used herbal formulas for diabetes according to previous research from NHIRD (Huang et al., 2013), were not noted in this study. Only a few patients were co-prescribed with derivative formulas, including Zhi-Bo-Di-Huang-Wan (three in the high-dose and five in the low-dose RCEG groups) and Qi-Ju-Di-Huang-Wan (one in each group). None of the co-prescription patterns were statistically significant.

**Table 3:** Within-group comparisons between the high and low treatment response populations within the high-dose RCEG group.

	High-response	(*n* = 40)	Low-response	(*n* = 37)	*p*-value
	Median (min, max)		Median (min, max)		
Demographic characteristics					
Age (years)	59.5 (42.0, 78.0)	(*n* = 40)	62.0 (41.0, 81.0)	(*n* = 37)	0.35
Gender, male/Female	23/17	(*n* = 40)	18/19	(*n* = 37)	0.50^a^
Duration of DM (years)	9.5 (1.0, 28.0)	(*n* = 40)	8.0 (1.0, 27.0)	(*n* = 37)	0.69
No. Of OHA (0/1/2/3/4)	18/3/7/10/2	(*n* = 40)	17/8/4/5/3	(*n* = 37)	0.31^a^
Smoke (Y/N)	2/38	(*n* = 40)	4/33	(*n* = 37)	0.42^a^
Alcohol (Y/N)	3/37	(*n* = 40)	2/35	(*n* = 37)	1.00^a^
Insulin used (Y/N)	3/37	(*n* = 40)	3/34	(*n* = 37)	1.00^a^
Lipid lowering drug (Y/N)	11/29	(*n* = 40)	14/23	(*n* = 37)	0.47^a^
Anthropometric characteristics and vital signs					
Weight (kg)	66.2 (40.0, 97.0)	(*n* = 40)	68.0 (47.2, 121.6)	(*n* = 37)	0.89
BMI (kg/m2)	25.7 (16.2, 33.5)	(*n* = 40)	25.5 (19.6, 41.6)	(*n* = 36)	0.64
SBP (mmHg)	138.0 (105.0, 187.0)	(*n* = 35)	135.0 (110.0, 177.0)	(*n* = 33)	0.74
DBP (mmHg)	80.0 (40.0, 112.0)	(*n* = 35)	76.0 (42.0, 104.0)	(*n* = 33)	0.72
HR (beat/min)	84.0 (61.0, 107.0)	(*n* = 31)	78.5 (60.0, 111.0)	(*n* = 28)	0.02*
Laboratory data					
HbA1c (%)	9.5 (6.6, 13.9)	(*n* = 40)	7.9 (6.5, 12.5)	(*n* = 37)	0.002**
FPG (mg/dl)	172.0 (83.0, 353.0)	(*n* = 21)	151.0 (91.0, 275.0)	(*n* = 18)	0.83
2 hPG (mg/dl)	233.5 (96.0, 436.0)	(*n* = 14)	271.0 (99.0, 421.0)	(*n* = 8)	0.92
ALT (U/L)	21.0 (9.0, 100.0)	(*n* = 25)	41.5 (15.0, 106.0)	(*n* = 20)	0.008**
Cr (mg/dl)	0.8 (0.3, 8.0)	(*n* = 25)	0.7 (0.5, 1.4)	(*n* = 21)	0.43
Total cholesterol (mg/dl)	165.5 (86.0, 384.0)	(*n* = 22)	188.0 (152.0, 276.0)	(*n* = 17)	0.07
HDL-C (mg/dl)	40.0 (26.0, 106.0)	(*n* = 21)	49.0 (36.0, 82.0)	(*n* = 15)	0.03*
LDL-C (mg/dl)	97.0 (20.0, 153.0)	(*n* = 21)	121.0 (60.0, 202.0)	(*n* = 16)	0.02*
Triglycerides (mg/dl)	149.0 (33.0, 2,589.0)	(*n* = 22)	109.0 (45.0, 227.0)	(*n* = 17)	0.32
Average daily dose and treatment duration of RCEG					
RCEG average dose (g/d)	9.9 (4.6, 16.7)	(*n* = 40)	9.0 (6.0, 13.8)	(*n* = 37)	0.04*
RCEG treatment (days)	185.5 (49.0, 441.0)	(*n* = 40)	75.0 (21.0, 756.0)	(*n* = 37)	<0.001***
Average daily dose of commonly combined CHM extract granules					
Shen-ling-Bai-Zhu-San (g/d)	7.2 (2.0, 12.0)	(*n* = 38)	7.4 (0.9, 9.0)	(*n* = 34)	0.96
Ban-Xia-Xie-Xin-Tang (g/d)	3.0 (1.2, 4.5)	(*n* = 7)	3.0 (1.5, 3.0)	(*n* = 3)	0.81
Bu-Yang-Huan-Wu-Tang (g/d)	4.5 (3.6, 6.0)	(*n* = 6)	3.2 (2.0, 4.3)	(*n* = 2)	0.29
Gui-Lu-Er-Xian-Jiao (pc/d)	4.8 (4.0, 6.0)	(*n* = 4)	5.3 (3.7, 9.0)	(*n* = 7)	0.92
LipoCol Forte Capsules (pc/d)	2.0 (1.4, 2.0)	(*n* = 5)	2.0 (1.6, 2.0)	(*n* = 7)	1.00
San-Qi (g/d)	1.5 (1.0, 1.5)	(*n* = 12)	1.5 (1.5, 3.0)	(*n* = 11)	0.11
Cang-Zhu (g/d)	1.5 (1.0, 7.5)	(*n* = 7)	3.6 (0.1, 9.0)	(*n* = 7)	0.85

Based on their treatment response, patients in the high-dose RCEG group were divided into two groups for characteristic evaluation. Data are presented as the median (min, max).

^a^ Fisher’s exact test was used for the categorical variables. Continuous variables were evaluated by the Mann Whitney U test. Significant differences (*p < 0.05; **p < 0.01; ***p < 0.001) were observed between the two group in several variables, including the average daily dose and treatment duration of RCEG, and initial HR, HbA1c, ALT, HDL-C, and LDL-C. FPG, fasting plasma glucose; ALT, alanine aminotransferase; BMI, body mass index; Cr, creatinine; DBP, diastolic blood pressure; DM, diabetes mellitus; HbA1c, glycated hemoglobin; HDL-C, high-density lipoprotein cholesterol; HR, heart rate; LDL-C, low-density lipoprotein cholesterol; OHA, oral hypoglycemia agents; 2 hPG, 2-hour postprandial glucose; SBP, systolic blood pressure.

## Discussion

There are 128 chemical components in *Rhizoma Coptidis* (Ma et al., 2019), and the majority are alkaloids. Among these alkaloids, berberine is the main active constituent that provides the therapeutic effect, but could be toxic if overdosed (Ma et al., 2010; Ning et al., 2015; Ma et al., 2019). The anti-diabetic function is one of the pharmacological spectra of *Rhizoma Coptidis* that has been proven *in vitro* and *in vivo* (Meng et al., 2018; Ma et al., 2019; Wang et al., 2019). However, the dose, clinical effects and long-term usage of *Rhizoma Coptidis*, especially RCEG, remain poorly understood. The production of extract granules in the last century represents one of the most important developments in the history of Chinese medicine. It is challenging for a TCM doctor to establish and formulate standard guidelines to prescribe extract granules of CHM, including *Rhizoma Coptidis*, in a proper dosage, because there are diverse origins of CHM and extraction methods adapted by manufacturers. Unlike OHA, the recommended dosages of herbal extract granules from different pharmaceutical companies may not be available, correct, or even comparable. Indeed, as the ancients said, “the non-spreading secrets of traditional Chinese medicine lie upon the prescription dosage”. Regardless of the patient's weight, the daily dose of RCEG recommended by the manufacturer is between 0.6 and 1.8 g per day, which is much less than the effective dose shown in this study for the glycemic control. In this retrospective study, we evaluated the hypoglycemic effects in patients with type 2 diabetes receiving high-dose (≥0.08 g/kg/d) and low-dose (<0.08 g/kg/d) RCEG, manufactured by Sun Ten pharmaceutical company. This report illustrated that only RCEG in excess of 0.08 g/kg/d demonstrated therapeutic value in terms of anti-diabetic and anti-hypercholesterolemic effects for patients with type 2 diabetes.

A population-based survey in Taiwan revealed that 77.9% of patients with type 2 diabetes received TCM. However, only 13.9% of them utilized TCM to control diabetes (Huang et al., 2013). Most patients with type 2 diabetes utilized CHM merely to relieve their diabetes-related symptoms, rather to stabilize their glycemic level. In line with this, Liu-Wei-Di-Huang-Wan and its derivatives are frequently chosen by TCM doctors to treat diabetes (Huang et al., 2013), despite the hypoglycemic effects being limited. In addition, TCM doctors often treat diabetes based on different clinical syndromes and disease stages (Guo et al., 2014; Pang et al., 2015c). *Rhizoma Coptidis* is prescribed for heat-clearing in the early and middle stage of T2DM (Pang et al., 2015a; Pang et al., 2015c), and Liu-Wei-Di-Huang-Wan is used for the later stage to counteract the yin deficiency, with beneficial effects on diabetic nephropathy (Pang et al., 2015c; Lin et al., 2016). This implies that most of the patients with diabetes from NHIRD sought TCM treatment in order to alleviate diabetic complications at a later stage of the disease. Therefore, it is challenging to analyze the efficacy of CHM extract granules on glycemic control based on data from the NHIRD. This study was the first to explore the efficacy and dose response of RCEG for glycemic control from large-scale datasets.

As shown in Table 1, the high-dose group had a longer duration of diabetes and a higher level of HbA1c prior to RCEG treatment. To achieve better glycemic control, TCM doctors would prescribe high-dose RCEG for such patients. It is also possible that TCM doctors chose the low-dose RCEG to treat other symptoms instead of controlling the glycemic level. Importantly, a previous pilot clinical study reported that YH1, containing high-dose RCEG and SLBZS, can benefit patients with poorly controlled type 2 diabetes who have already been treated with three or more kinds of OHA (Huang et al., 2019). After 3 months of YH1 add-on treatment, there were significant reductions in HbA1c and the lipid profile, as well as a significant increase in HOMA-β. Here, this study evaluated the same parameters, including HbA1c and lipid profiles, for up to 60 weeks for patients with type 2 diabetes receiving RCEG treatment, with or without OHA/insulin therapy. Longer extended treatment and follow-up periods in the high-dose RCEG group still showed similar benefits in terms of HbA1c and total cholesterol. For patients with or without OHA/insulin therapy, high-dose RCEG can be administrated to enhance the glycemic control, allowing the patient to approach the targeted goal of HbA1c <7% and improving hyperlipidemia at the same time.

Regarding the safety concerns of RCEG, high-dose treatment for up to 60 weeks has been shown to be safe, without obvious hepatic or renal toxicity, which has previously been reported (Huang et al., 2019). In this study, the range of RCEG in the high-dose group was between 0.08 and 0.23 g/kg/d, with a median of 0.14 g/kg/d (Supplementary Material S2). There is approximately 45–50 mg of berberine in 1 g of RCEG (Supplementary Material S1). Therefore, the dose of berberine in the highest dosage of 0.23 g/kg/d of RCEG is 11.5 mg/kg/d, which is lower than the dose prescribed in an earlier clinical trial (Yin et al., 2008) and much less than the quantity derived from the LD50 of *Rhizoma Coptidis* found in previous toxicity studies (Ning et al., 2015; Wang et al., 2019). Nevertheless, *Rhizoma Coptidis* is classified as a bitter-cold herb and has side effects on the gastrointestinal (GI) tract (Yin et al., 2008; Pang et al., 2015a; Pang et al., 2015b; Huang et al., 2019). In general, TCM doctors prescribe more than two CHMs to treat patients, in order to increase the synergistic effects and offset the adverse effects of CHM. In this retrospective study from the CGRD, SLBZS was the most frequent formula prescribed together with RCEG to treat type 2 diabetes. The function of SLBZS is to relieve the GI discomfort caused by *Rhizoma Coptidis* (Huang et al., 2019). According to TCM theory, when prescribing herbs with robust cool or cold properties, such as RCEG, it is recommended to include SLBZS, San-Qi, Ban-Xia-Xie-Xin-Tang, or Cang-Zhu to soothe the GI tract (Wang et al., 2010; Zhao et al., 2013; Lv et al., 2017; Wang and Zheng, 2019). The protective effect of the second herb can also enable RCEG treatment at a higher dose and for longer duration. In addition, diabetes is associated with several macro- and micro-vascular diseases. To increase blood circulation and prevent vascular complications, a blood regulation formula, including San-Qi, LipoCol Forte Capsules, Bu-Yang-Huan-Wu-Tang, or Dan-Shen, is commonly co-prescribed for patients with type 2 diabetes (Figure 3) (Ng, 2006; Jia et al., 2019; Zhao et al., 2019; Zhu et al., 2019). Because type 2 diabetes mellitus frequently co-exists with osteoarthritis (Veronese et al., 2019), Gui-Lu-Er-Xian-Jiao is often given to support the kidney yin and yang for patients with diabetes. This herbal formula can mitigate chondrocyte degeneration, cartilage destruction, and articular pain (Tsai et al., 2014; Chou et al., 2018). The current study identified high-dose RCEG and other drugs that are often co-prescribed with it for type 2 diabetes. These combinations of different treatments could serve as a compass of prescriptions for future clinical research.

This retrospective analysis based on data from the CGRD has the following limitations. First, the number of subjects could be higher if a database of nationwide hospitals and clinics was available. Unfortunately, most Chinese medicine practitioners did not evaluate the hypoglycemic effect of Chinese medicine based on the laboratory data. This study will encourage Chinese medicine practitioners to follow up laboratory data to objectively evaluate the hypoglycemic efficacy of CHM alone or a specific combination of CHM with OHA/insulin treatment in the future. Second, a more detailed stratified analysis could be executed if the patient’s records were well documented and preserved at all time points. Third, due to the bitterness of RCEG, pill and tablet formats are more suitable for better drug compliance in long-term treatment (Pang et al., 2015a). Fourth, RCEG supplies of uniform quality and standard were guaranteed by a single pharmaceutical manufacturer in this study. However, the effective dosage and duration of RCEG produced by another pharmaceutical company might not the same, and therefore must be re-examined. Lastly, there are different disease stages and patterns, with multiple combinations of symptoms, in patients with type 2 diabetes, according to TCM theory. *Rhizoma Coptidis* is recommended to treat middle wasting-thirst and hyperactive stomach heat. Such symptoms include central obesity, thirst, polyphagia, yellow tongue coating, and a slippery and powerful pulse (Zhang et al., 2010; Tong et al., 2012). Thus, high-dose RCEG might not be applicable for all patients with type 2 diabetes. In this study, patients may effectively respond to RCEG treatment if they better tolerate a higher dose and longer duration of RCEG treatment. Coincidentally, patients in the high-dose group, starting with a higher HbA1c level, higher heart rates, and better liver function, also had a better outcome. It is necessary for TCM doctors to assess the pros and cons of high-dose RCEG, and prescribe it accurately, to achieve an anti-diabetes outcome and to minimize adverse effects.

## Conclusions

This study indicated that high-dose RCEG (≥0.08 g/kg/d) can improve HbA1c, FPG and total cholesterol and is safe for patients with type 2 diabetes with or without OHA/insulin therapy. For each patient, the best outcome with minimum side-effects relies on tolerance to the dosage and duration of RCEG treatment. In addition, two of the most frequently prescribed CHMs alongside RCEG are digestive/tonic/dampness draining formulas and blood regulation recipes. This research serves as an evidence-based guideline for TCM doctors to treat type 2 diabetes with the proper dosage of RCEG and co-prescribed CHMs, and also provides alternative treatment options for patients to achieve better glycemic control by taking a phytomedicine. In the future, a large-scale randomized clinical trial with long-term follow-up should be launched to confirm these preliminary results.

## Data Availability Statement

The raw data supporting the conclusions of this article will be made available by the authors, without undue reservation.

## Ethics Statement

The Ethics Review Board of Chang Gung Medical Foundation in Taiwan approved this research (IRB No. 201701817B0). Written informed consent for participation was not required for this study in accordance with the national legislation and the institutional requirements.

## Author Contributions

Conceptualization, methodology and data curation, Y-HH and G-HL; formal analysis, T-YH; HPLC/PDA analysis of RCEG, M-CL; writing—original draft preparation, Y-HH; writing—review and editing, Y-HH and C-TH; validation, L-YY and Y-HW; supervision, Y-HW. All authors have read and agreed to the published version of the manuscript.

## Conflict of Interest

The authors declare that the research was conducted in the absence of any commercial or financial relationships that could be construed as a potential conflict of interest.
