# Supplementary material for: Effective Dose of Rhizoma Coptidis Extract Granules for Type 2 Diabetes Treatment: A Hospital-Based Retrospective Cohort Study
Source: Front Pharmacol. 2021 Jan 25;11:597703. doi: 10.3389/fphar.2020.597703 (PMC7868566; doi:10.3389/fphar.2020.597703)
Supplement: Supplementary file 1 [file datasheet1.pdf]

Supplementary File 1. Chemical analysis and HPLC fingerprint of RCEG.

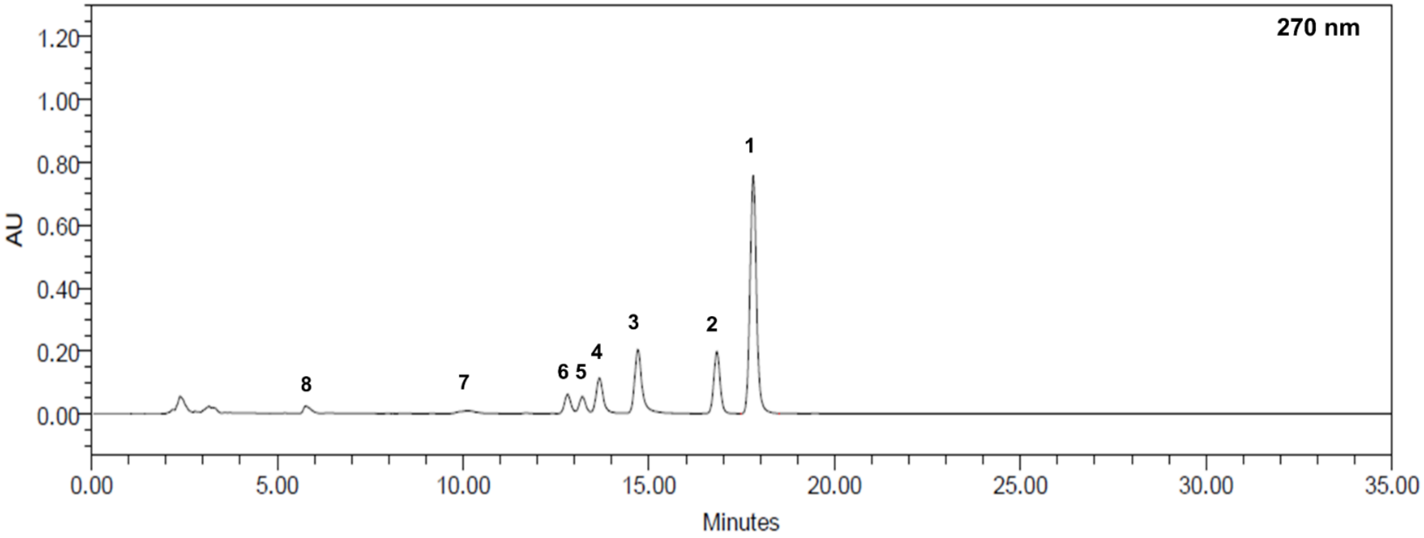

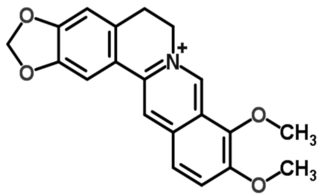

1. Berberine

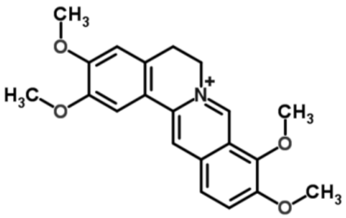

2. Palmatine

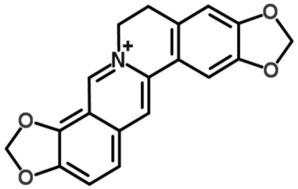

3. Coptisine

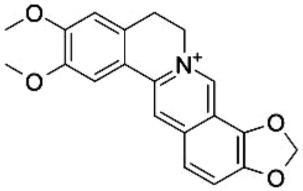

4. Epiberberine

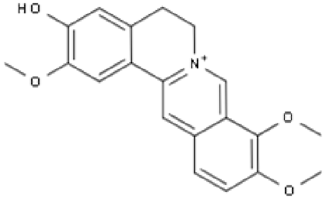

5. Jatrorrhizine

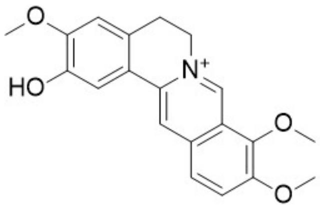

6. Columbamine

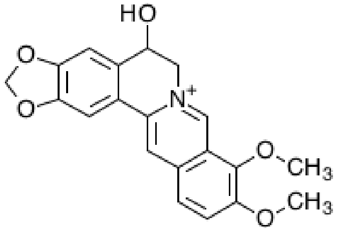

7. Berberastine

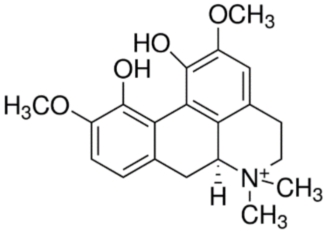

8. Magnoflorine

RCEG was produced by Sun Ten Pharmaceutical Co., LTD., a GMP manufacturer of herbal extract conforming to international standards. The chemical composition of *Rhizoma Coptidis* was analyzed using HPLC with photodiode array (PDA) detection. The sample preparation included mixing 0.1 g of *Rhizoma Coptidis* with 40 ml of methanol/water solution (70%/30%, v/v), ultra-sonicating at 25°C for 15 mins and then shaking at 160 rpm in a 40°C water bath for 20 mins. After centrifugation and filtration, 20 µl of sample solution was analyzed via HPLC. A Cosmosil 5C18-MS-II column was utilized as the stationary phase, and a gradient of mixture of sodium dodecyl sulfate (SDS), sodium acetate, acetic acid, acetonitrile, and water were used as the elute solution. The UV detection wavelength was set at 270 nm. The analytical run time was 35 mins. Eight peaks representing berberine, palmatine, coptisine, epiberberine, jatrorrhizine, columbamine, berberastine and magnoflorine were numbered in the chromatograms and their chemical structures were displayed accordingly. Berberine is the indicative compound of *Rhizoma Coptidis*. The following table summarizes the batch numbers of RCEG used in Chinese medicine clinics in the Taipei, Linkou, and Taoyuan branches of Chang Gung Memorial Hospital from January 1, 2008 to November 30, 2017. The berberine of RCEG in different batches from the same manufacturer were quantified among 45 to 50 mg/g. HPLC, high performance liquid chromatography; RCEG, *Rhizoma Coptidis* extract granules.

Years	2008	2009	2010	2011	2012	2013	2014	2015	2016	2017
Batch number of *Rhizoma Coptidis* extract granules	052914<br>163107<br>252410<br>272409<br>312011	023411<br>163107<br>183410<br>184406<br>203708<br>314410	055306<br>114508<br>114912<br>134906<br>235206	040108<br>060508<br>060509<br>070105<br>230309<br>235611<br>245612<br>275607	050805<br>050806<br>080810<br>131602<br>131610<br>141606<br>231207<br>271905	13020107<br>13020108<br>13052909<br>13052911<br>13053006<br>13083007<br>271905<br>301909<br>301910	13083007<br>14011703<br>14011704<br>14011706<br>14041003<br>14041009<br>14041106<br>14041107<br>14081409<br>14081410<br>14081507	14081508<br>14121811<br>14121907<br>15041411<br>15041508<br>15041603<br>15042007<br>15090310<br>15090909<br>15091003	15091003<br>16012009<br>16012204<br>16012506<br>16012507<br>16061303<br>16062709<br>16062710<br>16062807	16103109<br>16110110<br>16110209<br>17032106<br>17032107<br>17032209<br>17062108<br>17062109<br>17062206
