# Supplementary material for: Effective Dose of Rhizoma Coptidis Extract Granules for Type 2 Diabetes Treatment: A Hospital-Based Retrospective Cohort Study
Source: Front Pharmacol. 2021 Jan 25;11:597703. doi: 10.3389/fphar.2020.597703 (PMC7868566; doi:10.3389/fphar.2020.597703)
Supplement: Supplementary file 2 [file datasheet2.pdf]

Supplementary File 2. Dose-response effects of RCEG on relative change in HbA1c.

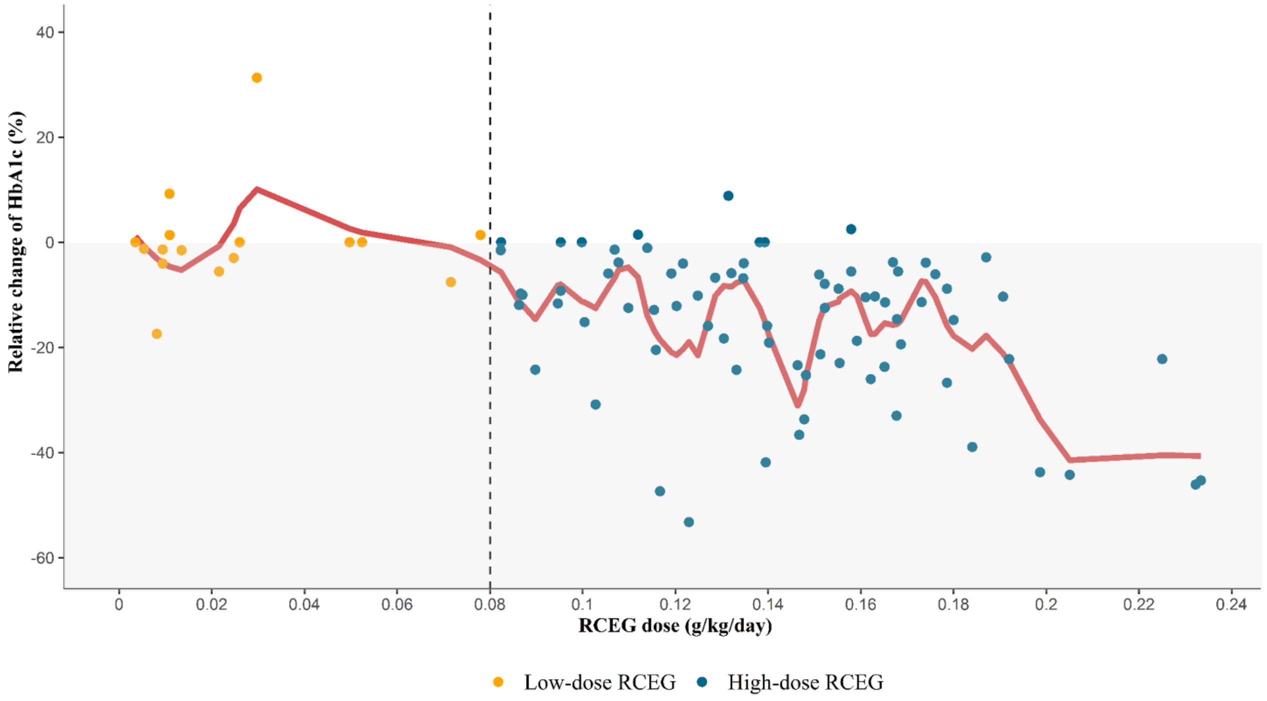

The correlation between RCEG dose and relative change of HbA1c was shown in scatter plots. Data from 93 subjects were analyzed by using the LOESS method. Relative change of HbA1c close to 0% representing a low-degree variability of treatment response was observed when RCEG dose is less than 0.08 g/kg/day. A significant relationship between RCEG dose and HbA1c reduction was found from 0.08 to 0.24 g/kg/day of RCEG. Thus, 0.08 g/kg/day of RCEG was set as a cut-off value to divide patients into the high-dose and low-dose RCEG group, presenting in aquamarine and orange dots, respectively. HbA1c, glycated hemoglobin; RCEG, *Rhizoma Coptidis* extract granules.
