# Supplementary material for: Effective Dose of Rhizoma Coptidis Extract Granules for Type 2 Diabetes Treatment: A Hospital-Based Retrospective Cohort Study
Source: Front Pharmacol. 2021 Jan 25;11:597703. doi: 10.3389/fphar.2020.597703 (PMC7868566; doi:10.3389/fphar.2020.597703)
Supplement: Supplementary file 3 [file datasheet3.pdf]

Supplementary File 3. Commonly co-prescribed CHM extract granules for type 2 diabetes.

CHM	Dosage (gm/day) Median (min., max.)	Effects	TCM indications	Number of patients (%) (total n = 77)	Number of prescriptions (%) (total n = 636)
Huang-Lian (RCEG)	9.5 (4.5, 16.6)	Cool heat, drain fire, clear damp-heat, detoxify and disinfect	Febrile illnesses, heart fire, stomach fire, jaundice, dysenteric disorders, diarrhea, infections in the middle burner	77 (100.0)	636 (100.0)
Shen-Ling-Bai-Zhu-San	7.3 (0.9, 12.0)	Tonify qi and fortify the spleen, drain dampness and harmonize the stomach	Spleen-stomach weakness, poor appetite, and sloppy stool	73 (94.8)	490 (77.0)
San-Qi	1.5 (0.8, 3.0)	Restrain blood, stop bleeding, break up blood stagnation, break up swelling, relieve pain	Bleeding, swelling, haematomas, contusion, traumatic injuries, abdominal pain, joint pain	28 (36.4)	130 (20.4)
LipoCol Forte Capsules[#]	2.0 (1.4, 2.0)	Promote digestion and blood circulation, tonify spleen and dry stomach	Hypercholesterolemia, hypertriglycemia	12 (15.6)	87 (13.7)
Ban-Xia-Xie-Xin-Tang	3.0 (1.2, 4.5)	Harmonize the stomach to downbear counterflow	Cold damage induced by early purgation, stuffiness and fullness below the heart, vomiting and borborigmus	10 (13.0)	62 (9.8)
Bu-Yang-Huan-Wu-Tang	4.4 (2.0, 6.0)	Tonify qi, active blood to free the collateral vessels	Hemiplegia, deviated eye and mouth, sluggish speech and sequela of wind stroke	8 (10.4)	62 (9.8)

Gui-Lu-Er-Xian-Jiao*	5.3 (3.7, 9.0)	Tonify the essence-marrow, tonify qi and nourish spirit	Consumptive disease, lumbago, seminal emission, impotence, enuresis, infertility, hypoadrenocorticism, sexual dysfunction, degeneration arthritis, and osteoporosis	11 (14.3)	57 (9.0)
Cang-Zhu	2.3 (0.1, 9.0)	Dry dampness, tonify and regulate the qi, disperse wind-dampness	Nausea and vomiting, epigastric pain, leucorrhoea painful obstruction disorder of the joints and limbs	14 (18.2)	55 (8.6)
Xiang-Sha-Liu-Jun-Zi-Tang	3.0 (1.0, 4.7)	Fortify the spleen and nourish the stomach	Qi deficiency and phlegm-retained fluid, stuffiness and oppression in vomiting, abdominal distention	8 (10.4)	41 (6.4)
Xiao-Qing-Long-Tang	0.8 (0.5, 2.0)	Release the exterior to dissipate cold, warm the lung and resolve fluid retention	Wind-cold induced by exopathogen, internal stagnation of fluid-dampness, aversion to cold with fever, absence of sweating, cough and panting, white and watery phlegm	8 (10.4)	30 (4.7)
Dan-Shen	1.3 (0.3, 1.5)	Move blood, break up blood stasis, cool heat, cool blood, calm and anchor the spirit	Stagnation, blood stasis, palpitations, insomnia, anxiety, restlessness, irritability	6 (7.8)	28 (4.4)

San-Zhong-Kui-Jian-Tang	5.5 (2.3, 6.0)	Clear heat and detoxicate, break accumulation and promote rupture	Scrofula and subcutaneous node	8 (10.4)	26 (4.1)
Suan-Zao-Ren-Tang	3.0 (2.0, 3.7)	Nourish the blood and tranquilization, clear heat and relieve restlessness	Irritability, anxiety, night sweats, fidgeting, sleep disturbance	5 (6.5)	22 (3.5)
Bai-Hu-Jia-Ren-Shen-Tang	6.1 (3.0, 7.8)	Clear heat, replenish qi and harmonize the middle	Dual damage of fluid and qi, polydipsia, summer heat stroke, fever and thirst, sweating and aversion to cold	5 (6.5)	22 (3.5)
He-Huan-Pi	3.0 (1.0, 3.0)	Calm and anchor the spirit, tonify blood, move the qi, spread liver qi	Insomnia, irritability, nervousness, agitation, anxiety, fear, insomnia, depression	11 (14.3)	20 (3.1)
Ye-Jiao-Teng	2.8 (1.5, 3.0)	Tonify blood, relieve pain, calm and anchor the spirit, unblock the channels and open the orifices	Agitation, irritability, insomnia, restlessness, numbness, pain in the limbs, paraesthesias, blood deficiency, liver deficiency	10 (13.0)	17 (2.7)
Jia-Wei-Xiao-Yao-San	2.0 (1.0, 2.0)	Soothe the liver and release depression, clear heat to cool the blood	Liver depression, blood deficiency and fever, menstrual irregularities, disquieted fearful throbbing	4 (5.2)	14 (2.2)
Yin-Chen	1.0 (0.9, 1.5)	Clear damp-heat, cool	Jaundice, constipation,	4 (5.2)	13 (2.0)

		heat	hepatitis, cholelithiasis, cholecystitis, intermittent fever, loss of appetite, bitter taste in the mouth		
Ma-Xing-Gan-Shi-Tang	4.6 (1.9, 9.0)	Diffusion with pungent-cool, clear the lung to calm panting	Pathogenic heat congesting the lung, fever, cough and panting	4 (5.2)	13 (2.0)
Gan-Jiang	0.6 (0.3, 0.9)	Warm the yang, disperse cold, dispel damp-cold, transform cold phlegm	Interior cold, especially cold in the spleen (vomiting, diarrhea, nausea, feeling of cold) and lung (cough, thin white sputum)	6 (7.8)	9 (1.4)
Ling-Gui-Zhu-Gan-Tang	3.0 (1.0, 6.0)	Fortify the spleen and drain dampness, warm and resolve phlegm	Phlegm-retained fluid disease, fullness and distention in the chest and hypochondrium, dizziness and palpitations, shortness of breath and cough	5 (6.5)	9 (1.4)
Yi-Gan-Sang	2.0 (2.0, 6.0)	Clear liver heat	Dampness-heat in the liver meridian, fright palpitations and convulsions, vomiting and phlegm drool, distention and fullness in abdomen, poor appetite, insomnia	4 (5.2)	6 (0.9)
Cang-Er-San	3.0 (2.0, 3.0)	Disperse wind to clear heat	Sinusitis (persistent excessive flow of turbid nasal discharge)	4 (5.2)	4 (0.6)

CHM, Chinese herbal medicines; RCEG, *Rhizoma Coptidis* extract granules

# Each LipoCol Forte Capsules contains 0.6 gram of red yeast rice.

* Formulation of Gui-lu-er-xian-jiao was prescribed as a pill weighted 0.5 gram per piece.
